# Preliminary In Vitro Assessment of Whey Protein Isolate Hydrogel with Cannabidiol as a Potential Hydrophobic Oral Drug Delivery System for Colorectal Cancer Therapy

**DOI:** 10.3390/polym16233273

**Published:** 2024-11-24

**Authors:** Daniel K. Baines, Karen Wright, Timothy E. L. Douglas

**Affiliations:** 1 School of Engineering, Lancaster University, Gillow Avenue, Lancaster LA1 4YW, UK; d.baines3@lancaster.ac.uk; 2 Biomedical and Life Sciences, Lancaster University, Gillow Avenue, Lancaster LA1 4YW, UK; karen.wright@lancaster.ac.uk

**Keywords:** whey protein, cannabidiol, β-lactoglobulin, cancer therapy, swelling, enzymes, oral medication, intravenous, hydrophobic drug delivery

## Abstract

Colorectal cancer (CRC) is the second global cause of cancer morbidity. Often, potent CRC drugs fail to reach the market, due to the molecule having low solubility levels. Therefore, there is a need to develop a viable, targeted delivery system for hydrophobic drugs. Whey protein isolate (WPI), in the form of hydrogels, has demonstrated loadability with hydrophobic molecules. Hydrophobic cannabidiol (CBD) has demonstrated potential in inhibiting and suppressing CRC tumour growth. Therefore, in this study, WPI hydrogels were assessed as a novel oral hydrophobic drug delivery vehicle, using CBD as a model drug. The hydrogels were analysed in conditions consistent with the alimentary tract. The investigation was performed at pH 2 (stomach), pH 7 (small intestines) and pH 9 (large intestines) and using the enzymes pepsin (stomach) and protease (small and large intestines) to simulate the digestive environment. Polymer swelling assays demonstrated that the swelling potential of the hydrogels was strongly dependent on pH. At pH 2, hydrogels decreased in mass, losing around 10% of their initial mass, while hydrogels in a pH 9 environment increased in mass by approximately 50%. However, the enzymatic degradation of the hydrogels at pH 2 (pepsin, stomach), pH 7 (protease, small intestines) and pH 9 (protease, large intestines) was more pronounced in the neutral–alkaline pH range. Pepsin at pH 2 had no significant effect on the hydrogels. In contrast, protease at pH 9 significantly degraded the hydrogels, resulting in a mass loss of 30–40% from the initial mass. The results suggesting a higher rate of degradation in the intestines rather than in the stomach. Furthermore, CBD release, analysed with U.V. spectroscopy, demonstrated a higher release rate in pH conditions associated with the intestines (pH 7 and pH 9) rather than the stomach (pH 2), suggesting a higher rate of CBD release in regions of the digestive tract affected by CRC. Significantly, the hydrogels significantly reduced the viability of HT29 CRC cells. This study demonstrates the potential of the utilisation of WPI hydrogels as an oral hydrophobic drug delivery system.

## 1. Introduction

Colorectal cancer CRC, also referred to as bowel cancer, is a form of cancer which begins as polyps in the colon before tumour formation and metastasising to other areas of the body [1]. Approximately 11% of all new cancer cases are attributed to CRC, with over 44,000 new cases reported in Britain in 2019 [2]. Globally, CRC is the second leading cause of cancer morbidity [3]. Familial inheritance and lifestyles risks have been associated with CRC development [4,5,6].

Currently, the gold standard CRC treatment is surgery to remove the polyps and the cancerous cell from the epithelial lining [7]. Chemotherapy, immunotherapy and radiotherapy are also utilised in conjunction [8]. A limitation presented by many potential CRC therapeutics ascends from their hydrophobic nature [9]. The hydrophobicity of the drug leads to poor solubility and poor bioavailability and presents difficulties in drug administration [10]. The result is a dependence on less potent medications or an increasing cost on the medications that succeed in passing the clinical trial phase [11]. To bring a typical cancer drug onto the market, an average of $648 million dollars is required. This cost is further increased by the number of medications that fail to reach the market beforehand [12]. Here, in this study, we suggest a potential safe and inexpensive solution to the delivery of hydrophobic medications to the target site using whey protein isolate (WPI) hydrogels, specifically utilising WPI hydrogel as an oral drug delivery system.

**Figure 1:**
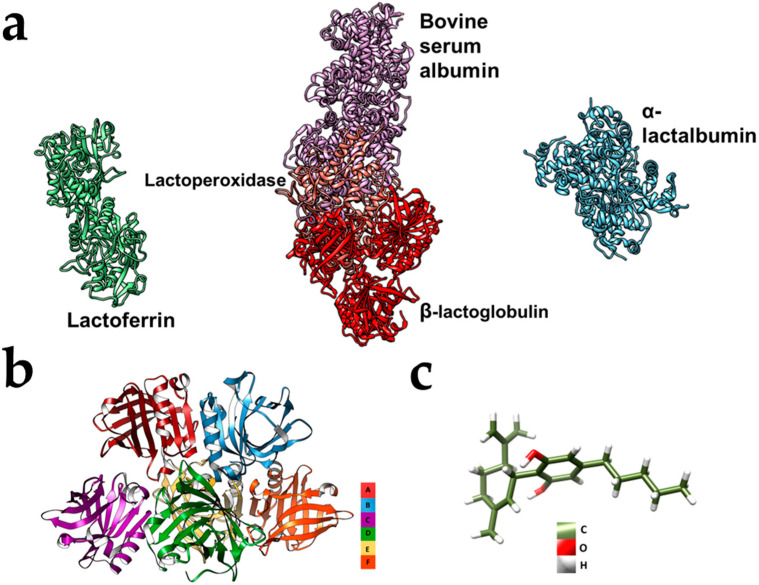
(**a**–**c**) Chimera models representing (**a**) the binding or non-binding of the 5 main constituent proteins in WPI in their functional form. (**b**) β-lactoglobulin and (**c**) a CBD molecule. The models were produced on Chimera (University of San Francisco, San Francisco, CA, USA). The WPI proteins were sourced from Uniprot (NCIB), and the CBD structure was sourced from PubChem (NCIB).

Recent advancements in the field of biomaterials have demonstrated the potential of WPI (Figure 1) [13]. Hydrophobic molecules can be incorporated into WPI hydrogels; this is believed to be thanks to hydrophobic interactions [14]. The hydrogels are easily sterilizable by autoclaving and their utilization as an oral drug delivery system presents no immediate risk. On the contrary, WPI is used as a dietary supplement, e.g., in milkshakes for bodybuilders. WPI hydrogels have been utilised in bone regenerative medicine with many different phases incorporated into the hydrogels [15,16,17]. However, it is the potential to incorporate poorly soluble molecules such as phloroglucinol that will be exploited in this investigation [18]. The hydrophobic phyto-cannabinoid, cannabidiol (CBD), Figure 1c, was the model drug in this investigation. CBD provided the ideal model as it is hydrophobic, inexpensive and easily available. Although the pharmacokinetics of CBD are not well defined [19], CBD has demonstrated positive properties, such as inhibiting the proliferation of tumours specifically in CRC both in vitro and in vivo [20]. Weng et al. [21] demonstrated that the elevated ROS levels caused by CBD inhibited tumour growth in p53wt cells through the arrest of cells in the G0/G1 phase of the cell cycle, initiating macroautophagy. Furthermore, ref. [22] demonstrated the inhibition of metastasis of CRC cells with CBD achieved through the inhibition of Wnt/β-catenin signalling pathway.

The protein-based nature of the WPI hydrogels presents a limiting factor in the use of WPI hydrogels as a hydrophobic drug delivery system, mainly due to the pH and proteolytic enzymes employed by the digestive system. The alimentary tract presents a varying range of pH values. The stomach demonstrates a range of pH 1.5–3.5 as a result of hydrochloric acid [23]. However, as bile and pancreatic secretions are introduced, and the partially digested produce enters the small intestine, the pH increases and can range from neutral to pH 8.5, before returning to neutral in the large intestine [24]. Additionally, digestion introduces the hydrogel to digestive enzymes, the most prevalent in the stomach being pepsin, whereas the intestines employ a range of proteolytic, pancreatic enzymes, released to aid in digestion [25]. 

Therefore, WPI hydrogels loaded with CBD (WPI-CBD) were synthesized with five different CBD concentrations (Table 1) and analysed physio-chemically in conditions consistent with the alimentary tract, specifically, at pH levels consistent with the stomach and the intestines. Enzymatic degradation assays were conducted using the digestive enzymes pepsin and proteases at concentrations consistent with digestion to ascertain any potential proteolytic effects. The release of CBD from the hydrogel was analysed utilising U.V. vis spectroscopy. Additionally, cell viability assays were conducted to analyse the effect on HT29 CRC.

## 2. Materials and Methods

### 2.1. WPI/CBD Hydrogel Formation

Whey Protein isolate (WPI) hydrogel samples were prepared to a concentration of 40% WPI *w*/*v* with Milli-Q water. The WPI was sourced from Davisco, foods international, Le Sueur, MN, USA. Cannabidiol (CBD) source from Tocris (Bristol, UK) was added to the concentrations of 10, 20, 30, 40 and 50 μM. The samples were vortexed for 10 s. Full homogenisation was achieved utilising an IKA Loopster (Oxford, UK) for 24 h. The solutions were degassed in a vacuum chamber. Gelation was achieved through heat induction in a water bath at 70 °C. Sterilisation was achieved through autoclaving.

### 2.2. Swelling Analysis

The assay utilized pH 2, pH 7 and pH 9 to investigate a range of pH levels in the digestive process. The investigation introduced 1 g WPI-CBD hydrogels to 5 mL of solutions at pH 2, pH 7 and pH 9. The samples were incubated at 37 °C. The mass of the hydrogel was taken gravimetrically at the start point and the end point of 5 days. The ratio mass percentage S% was calculated to the equation where Mw is equal to the wet mass and Md the dry mass.

S% = (Mw − Md)/Md × 100

### 2.3. Enzymatic Degradation Analysis

Two enzymatic degradation assays were performed to introduce WPI-CBD hydrogels to an enzymatic digestive associated enzyme. CBD-WPI hydrogels samples were formed to the concentrations consistent with the investigation to the mass of circa 1 g. The hydrogels were introduced 5 mL solution pepsin at pH 2 or protease at pH 7 and pH 9 formed to concentrations consistent with [26]. The samples were incubated at 37 °C for 5 days. The start and end masses were recorded gravimetrically. The swelling percentage S% was calculated using the equation where Mw is equal to the wet mass and Md the dry mass.

S% = (Mw − Md)/Md × 100

### 2.4. Release Profiling

The use of any targeted drug delivery mechanism requires the release of the medication at the target site. An assay was developed to ascertain the release of CBD from the WPI hydrogels. The assay determined the release of CBD at pH values 2, 7 and 9. The variables were chosen as they correlated with the pH values present in the digestive tract. The investigation was developed by adapting a method from [26]. Here, 1 g WPI-CBD samples were introduced to 5 mL of the relevant solution. The samples were incubated at 37 °C. At 24 h timepoints, 100 µL was removed from each sample and replaced with 100 µL of fresh solution, for 5 days. The samples were analysed utilising U.V. visible spectroscopy with an emphasis on 273 nm the region of interest for CBD (*n* = 15). 

### 2.5. Cell Viability Assay

Cellular viability assay was conducted with a Human CRC HT29 cell-line. The cells were cultured in DMEM medium supplemented with 10% fetal bovine serum (FBS) and incubated at in a CO_2_ incubator at 37 °C. The cells were trypsinised and 0.5 mL DMEM containing cells at the concentration 20 × 10^−5^ was added to a well plate containing WPI-CBD hydrogels cut to 3 mm thickness. The hydrogels had been previously sterilised through U.V radiation, for 10 min. At time points 72 h and 96 h, the cells were stained with the Presto Blue dye and fluorescence was ascertained at λ 570/600 nm (*n* = 16).

## 3. Results and Discussion

### 3.1. Swelling Analysis Under Conditions Simulating the Gastrointestinal Tract

**Figure 2:**
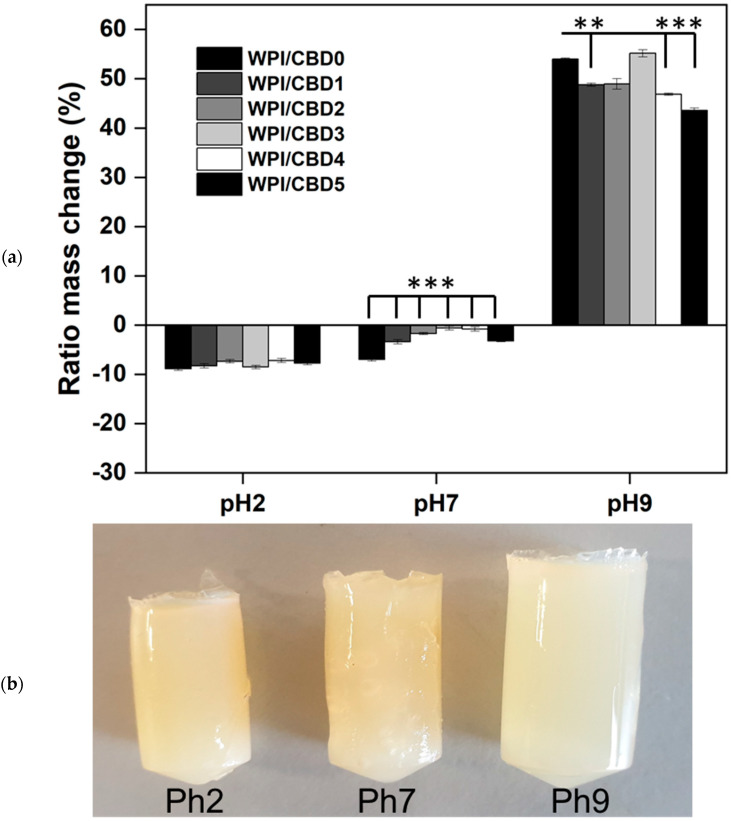
(**a**) The result of polymer swelling assay under pH 2, pH 7 and pH 9 conditions. The hydrogel samples were introduced to 5 mL solution. Each bar represents the mean ± SD of *n* = 15 (** *p* < 0.01, *** *p* < 0.001 when compared to the WPI control). (**b**) An image representing the hydrogels post swelling assay.

It was necessary to understand the behaviour of the hydrogels under physical conditions associated with digestion. For the assay, WPI and WPI-CBD hydrogels were introduced to environments at pH 2, pH 7 and pH 9. The results are presented in Figure 2. There are two main observations. Firstly, the addition of CBD affected the WPI hydrogel polymer swelling potential and secondly, increasing the pH caused increased swelling of the hydrogels.

In the acidic range, the addition of CBD had no significant effect on the swelling potential of the hydrogel when the samples containing CBD were compared to the WPI control without CBD. In contrast, the addition of CBD to the hydrogels led to statistically significant differences between the WPI-CBD concentrations for both pH 7 and pH 9 conditions, when compared to the WPI/CBD0 control. For instance, in the pH 7 environment, all WPI-CBD sample groups demonstrated statistically significant differences when compared to the WPI/CBD0 control (*p* < 0.05). Furthermore, the WPI/CBD0 control in the pH 7 environment aligns with previous WPI controls under pH 7 conditions, such as in Baines, et al. [15]. Similarly, when introduced to pH 9, the sample groups WPI-CBD1, WPI-CBD4 and WPI-CBD5 demonstrated statistically significant differences when compared the WPI/CBD0 control (*p* < 0.05). The WPICBD3 result in the pH9 solution could potentially be attributed to imperfections within the hydrogels, such as factures or air pockets formed during the manufacturing process, but such discussion must remain speculative.

The results suggested that the addition of CBD influenced the swelling potential of the hydrogels as demonstrated by two factors: Firstly, the decrease in mass loss at pH 7 groups with an increase in CBD concentration; secondly, the decrease in swelling at pH 9 with an increase in CBD concentration. The result could potentially be attributed to the underlying hydrophobic interactions between CBD and the WPI hydrogel proteins, affecting the solute uptake of the hydrogels. However, more investigation would be needed to verify this, due to the low concentrations of CBD utilised in the investigation.

The contrasting pH levels presented observable differences, as demonstrated in Figure 2. Acidic–neutral conditions resulted in a loss of mass that was more pronounced at the acidic portion of the scale. The relative mass loss improved, that is to say, less mass was lost as the environment became more neutral, demonstrated by the results for both the pH 2 and pH 7 values. In contrast, at the alkaline end of the scale, pH 9, the samples increased in mass significantly, a result of the different charges applied to the hydrogels during the swelling process. Betz et al. [27] suggested that the lack of swelling in low pH environments was attributed to a loss of carboxylic groups during the process of swelling. However, ref. [28] suggests that the main component of WPI, β-lactoglobulin in hydrogel form, was extremely stable in acid pH environments and suggests that the lack of swelling in low pH environment is partly due to the rigid structure of the β-lactoglobulin. Our findings suggest that both statements could be true. β-lactoglobulin is the main components of WPI. However, the concentration of β-lactoglobulin in WPI fluctuates between 60 and 90%; other proteins include, α-lactalbumin, bovine serum albumin, Lactotransferrin and Lactoperoxidase, amongst traces of other proteins. Therefore, there is potential for β-lactoglobulin to remain stable whilst other proteins’ carboxylic groups are deprotonated. Similarly, the effect attributed to swelling of the hydrogels under pH 9 conditions align with what is suggested in the literature. As suggested by Mercade-Prieto et al., the increase in swelling can be attributed to the interruption of intermolecular non-covalent interactions caused by the protein repulsion of the high number of charges, resulting if further protein deformation [29].

### 3.2. Enzymatic Degradation

**Figure 3:**
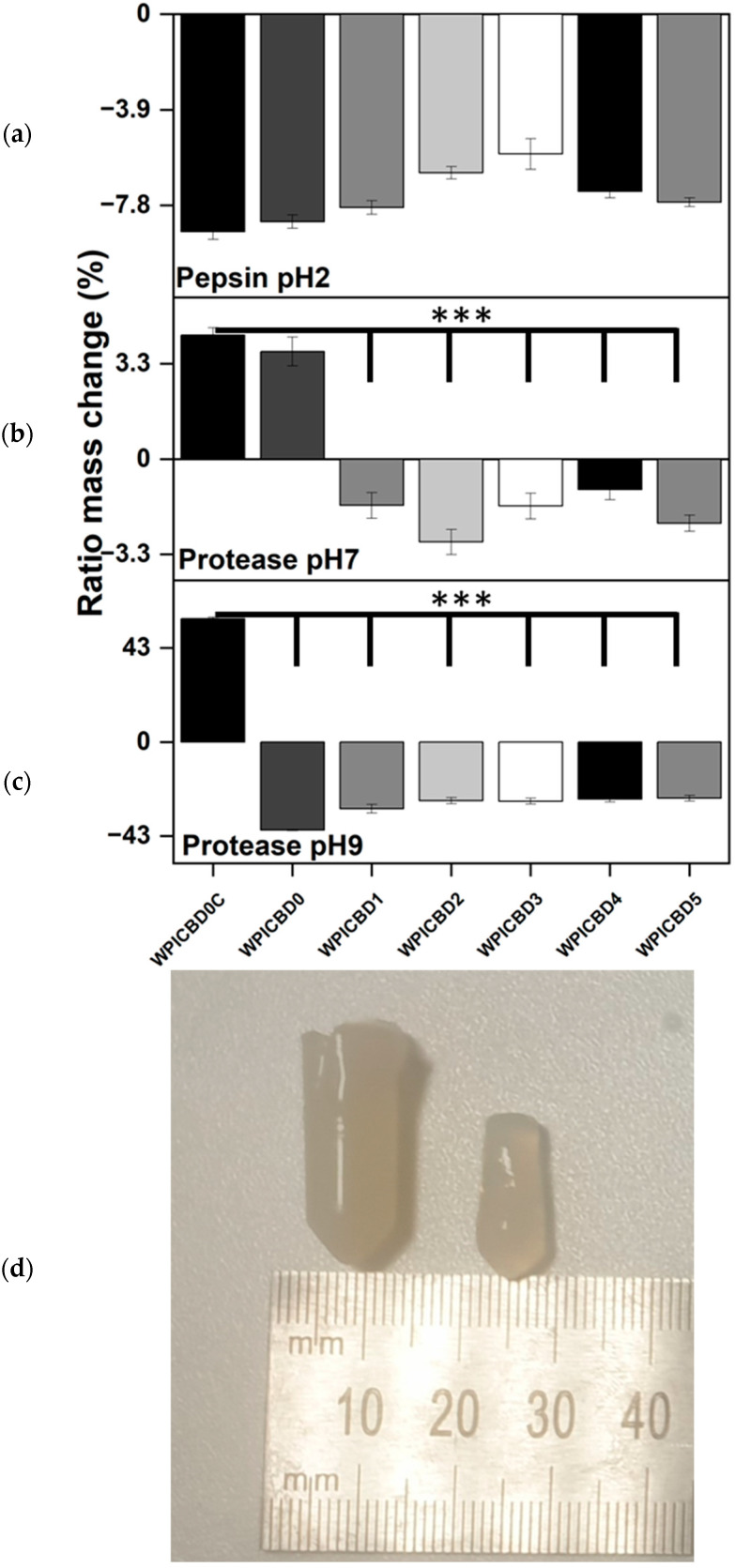
(**a**) Enzymatic degradation assay under (**a**) pH 2, (**b**) pH 7 and (**c**) pH 9 conditions. The hydrogel samples were introduced to 5 mL solution with added enzymes, (**a**) pepsin (pH 2) and (**b**) protease (pH 7 and pH 9). The WPICBD0C variant represents the control with no enzymes added to the solution, whereas WPICBD0 is the control with no added CBD. Each bar represents the mean ± SD of *n* = 10 (*** *p* < 0.001; compared to the WPI control). (**d**) an image depicts the pH9 WPICBD0C control with no enzymes (left) post swelling and degradation v the WPI0 control with enzymes (right).

Utilised as an oral medication, there is the potential for the protein-based hydrogels to be degraded through proteolysis by the enzymes of the digestive tract. Therefore, an assay was conducted to determine the effect of digestive enzymes on the degradation of the hydrogels. All enzyme concentrations were consistent with those found in the digestive track. Pepsin at pH 2 was used to replicate the stomach conditions, whilst protease was used to replicate proteolytic enzymes during digestion at pH 7 and pH 9. The results for the enzymatic degradation assays can be seen in Figure 3.

The results show a clear effect of proteolysis on the hydrogels, particularly under alkaline conditions. However, a clear observable difference was observed throughout the pH range. As suggested by the swelling assays at pH 2, the hydrogels would be expected to decrease in mass due to pH alone. Additionally, at pH 2, in the presence of pepsin at its optimal pH, a further loss of mass could be expected. However, this was not the case and marked differences were observed in the behaviours between the WPI hydrogels containing CBD and their control counterparts. The results may be a consequence of the lack of swelling, with the result that pepsin is not able to access the protein bonds which need to be cleaved in order to degrade the hydrogels.

Additionally, the observable results for the pH 7 and pH 9 values can be explained by both the results of the aforementioned swelling assay, with the enzyme gaining access to bonds thanks to swelling, and enzyme kinetics. In the assay, under basic conditions, the enzymatic effect of protease was much more pronounced. The hydrogels were degraded significantly when compared to the neutral environment. This may be the result of both the increased access to bonds gained by the protease during the swelling of the hydrogels in basic conditions and protease having an optimum pH of 8–9 [30]. At pH 9, significant differences were observed between the WPI hydrogels containing CBD and the CBD-free control without any enzyme. Likewise, at pH 7, significant differences were observed between the controls. However, the WPI/CBD0 control gained mass during the assay. WPI hydrogels in neutral conditions have the potential to gain or lose a small amount of mass. Therefore, the mass loss shown by the WPI-CBD variables could also be attributed to hydrogel variability. However, the pronounced effect of the enzyme on the hydrogel between acidic to neutral and neutral to basic suggests a system that suffers less degradation in conditions consistent with the stomach and could potentially be protected in the acidic conditions, before moving to conditions associated with the large and small intestines and beginning to degrade fully. Furthermore, the increased degradation in the intestines would increase the rate of the release of CBD from the hydrogel at the target site.

### 3.3. Release Process 

#### 3.3.1. Standardisation Process

##### β-Lactoglobulin and α-Lactalbumin Standardisation

For method standardisation, β-lactoglobulin and α-lactalbumin were chosen. β-lactoglobulin and α-lactalbumin are the two main protein components of WPI, accounting for between 60–90% (β-lactoglobulin) and 15–25% (α-lactalbumin) of the WPI [31]. Additionally, β-lactoglobulin and α-albumin present small-scale differences in their tyrosine (Tyr) and tryptophan (Trp) residue composition and both absorb at wavelengths associated with CBD absorbance due to Tyr, Trp and CBD all presenting molecular aromatic portions. 

**Figure 4:**
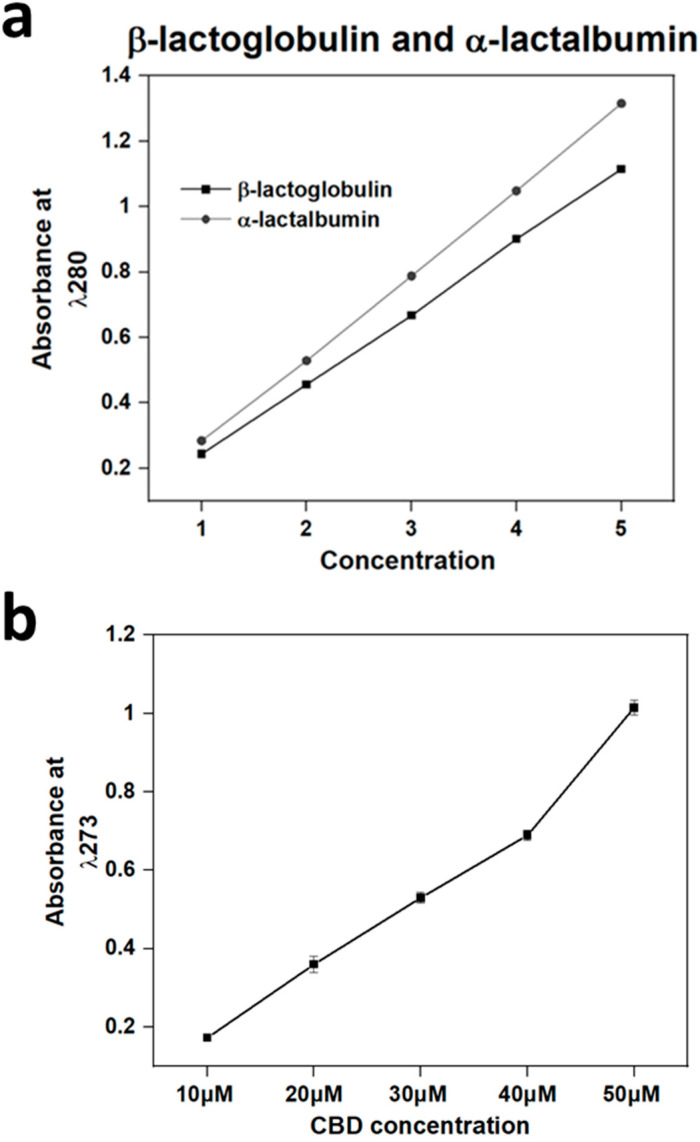
(**a**,**b**): (**a**) Release standardisation assay with U.V absorbance of α-lactalbumin and β-lactoglobulin (10^−4^ g/M/L) at λ280 nm. The maximum concentration used was 10^−4^ g/M/L, labelled in graph a as 5. (**b**) CBD calibration curve with a maximum concentration of 50 μΜ, with U.V absorbance of CBD at λ273 nm. Each bar represents the mean ± SD of *n* = 15.

The results displayed in Figure 4a demonstrate a linear increase for both β-lactoglobulin and α-lactalbumin from the minimum concentration to the maximum concentration of 10^−4^ g/M/L. Additionally, as expected, α-lactalbumin presented a higher absorbance in the λ280 nm region due to the increased number of Tyr and Trp residues in the α-lactalbumin amino acid composition. 

##### CBD Calibration

The results for the CBD calibration curve are displayed in Figure 4b and Table 2. A linear increase in CBD is demonstrated between the concentrations 10, 20, 30 and 40 μΜ. However, the 50 μM variable deviates from the trend, displaying a higher absorbance than the trend, suggesting the beginning of an oversaturation of aromatic rings.

##### CBD Release Profiling

**Figure 5:**
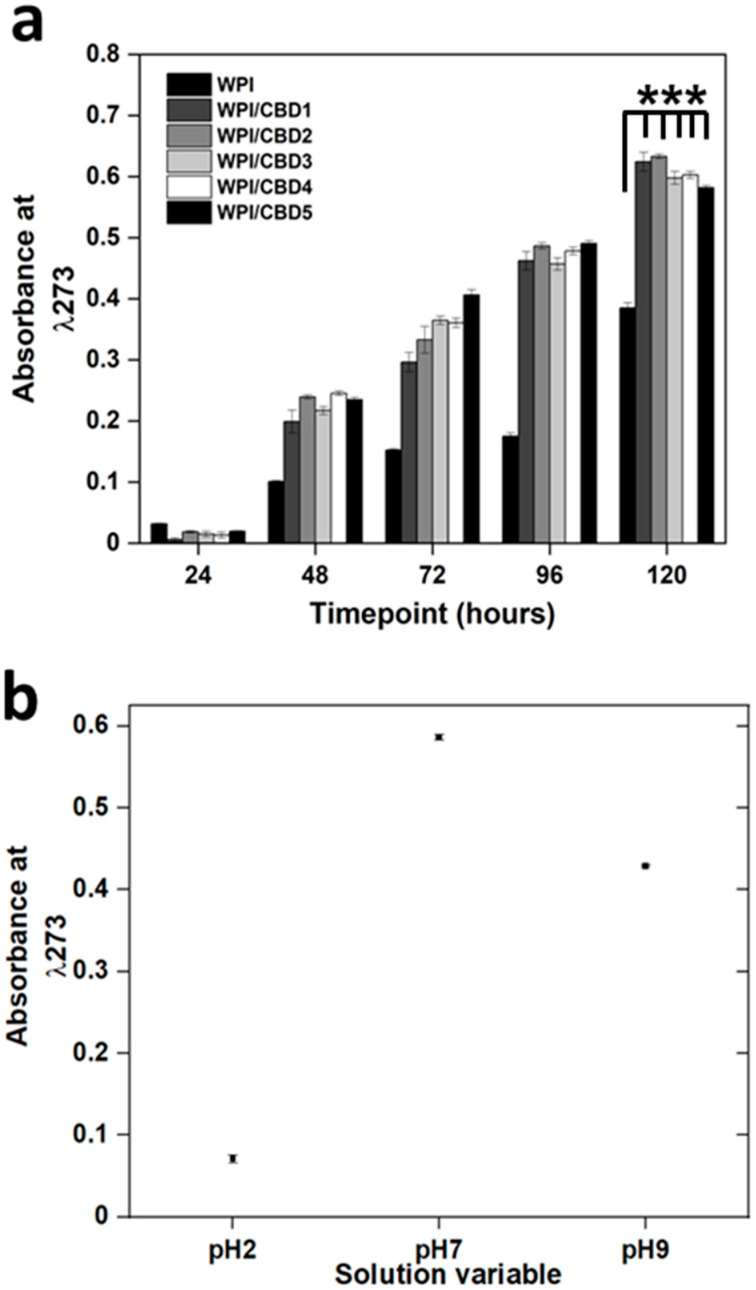
(**a**,**b**): (**a**) The observables are a, example CBD release in PBS, and (**b**), the relative absorbance of U.V radiation at λ273 nm for the release of CBD at the end point concentration (WPI/CBD5) in each pH variable. Each bar represents the mean ± SD of *n* = 15 (*** *p* < 0.001 when compared to the WPI control).

Release analysis was performed on the WPI-CBD hydrogels. WPI hydrogels may bind hydrophobic molecules through hydrophobic interaction on the inside of the hydrogels. Therefore, it was important to ascertain if the release of CBD from the hydrogel was possible, but also if release was possible in conditions consistent with digestion. The cumulative release was monitored over 5 days in pH 2, PH 7 and pH 9 solutions. The results are presented in Figure 5a,b.

The results in Figure 5a demonstrate the release of CBD over a 5-day period at pH 7. The results suggest a significant difference in absorbance between the WPI-CBD sample groups and the WPI/CBD0 control (*p* < 0.05). Further analysis at 120 h suggests the potential CBD release of around 10–15 μM when compared to Table 1. A 2022 review from Heider et al. [32] assessed the known CBD anti-cancer effects and cited seven independent CRC investigations where CBD had produced inhibited tumour proliferation at concentrations under 15 μM. For instance, Aviello et al. [33] inhibited cellular proliferation in Caco-2 and HCT116 cell lines with 10 μM CBD. Jeong et al. [34] increased apoptosis in HCT116 and DLD1 cell lines with 6 μM CBD. Furthermore, Kim et al. [35] demonstrated increased apoptosis in HCT116, DLD-1 and HT29 CRC cell lines with 4 μM CBD. These all suggest the potential of WPI-CBD hydrogels should release occur at the target site.

When comparisons are made between the pH 7 values and both the pH 2 and pH 9 values in Figure 5b, a maximum release is at pH 7 is observable. The samples fail to release CBD under pH 2 conditions. Furthermore, no protein leaching can be observed, further demonstrating the molecular effect of pH 2 on the hydrogels as aforenoted in the swelling and enzymatic degradation sections. The release at pH 9 equates to approximately 5–10 μM. Although lower than at pH 7, the release at pH 9 still equates to a concentration that could have positive results. The results suggest that maximum CBD release would occur in pH conditions consistent with those in the intestines. Additionally, the release assay was conducted without any enzyme present. Therefore, once in the digestive tract and introduced to digestive enzymes, both the rate and the concentration of release would be increased. However, the maximum release would be in conditions consistent with the intestines and, thus, the target site.

### 3.4. Cellular Analysis

#### Cell Viability

**Figure 6:**
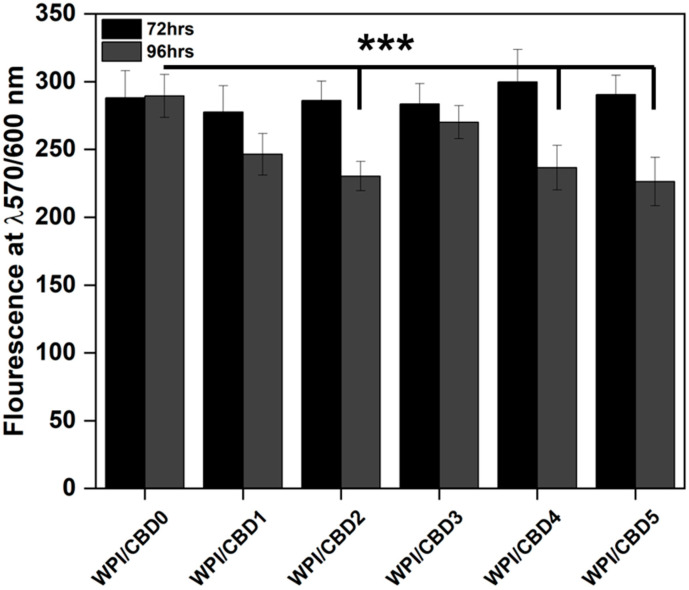
WPI-CBD hydrogel cellular viability results on HT29 cells. Black depicts cellular viability post 72 h and dark grey 96 h. The Y axis shows the florescence at 570/600 nm and the X axis depicts the WPI-CBD hydrogels in increasing concentrations, demonstrated in Table 2. Each bar represents the mean ± SD of *n* = 16 (*** *p* < 0.001 when compared to the WPI control).

CBD has previously been shown to have an antiproliferative effect on HT29 cells in vitro [33]. In order to ascertain whether WPI-CBD gels would be able to impact on cell viability, HT29 cells were treated for 72 and 96 h (Figure 6). CBD had no effect up to 72 h but was able to reduce cell viability by 20.4% (WPI-CBD2), 18.3% (WPI-CBD4) and 21.8% (WPI-CBD5) (*p* < 0.05).

Although full apoptosis was not observed, the results demonstrate a reduction in proliferation between timepoint 72 h and 96 h. When compared to the WPI/CBD0 control variable, WPI-CBD2, WPI-CBD4 and WPI-CBD5 demonstrate significant differences, suggesting the potential role of CBD. Sainz-cort et al. [36] demonstrated the anti-proliferative effect of CBD on HT29 cells at a concentration of 10 μM. In their investigation, at 48 h, the HT29 cellular proliferation was significantly lower than that of the control variables. However, full cytotoxicity was not observed but anti-proliferative effects were. Although there is a 2-day discrepancy between the observable anti-proliferation in this assay and the one conducted by [37], the discrepancy is likely the result of CBD encapsulation in the hydrogel and, thus, the time required for the consequent CBD release. Similar cytotoxic and anti-proliferative effects of CBD on HT29 CRC cells have been observed by [38,39,40].

The results provide further validity to the previous CBD release assay. Furthermore, the results demonstrate the potential of WPI hydrogels to encapsule, protect and deliver a hydrophobic molecule to fulfil its medicinal role.

## 4. Conclusions

The investigation sought to synthesis WPI hydrogels with a known hydrophobic molecule; in this study, CBD was utilised. The novel approach was taken due to the benefits provided by WPI. It is known that hydrophobic molecules can be incorporated in WPI hydrogels. Further benefits are the low cost of WPI, which can potentially be exploited to produce a cheaper alternative for hydrophobic drug delivery. A further part of the investigation was the analysis of the hydrogels in conditions consistent with human digestion to determine if the material would be valuable for further analysis to provide a hydrophobic drug delivery system to treat CRC.

The first conclusion is the successful incorporation of hydrophobic CBD into the WPI hydrogels. The results suggest that CBD was encapsulated in the WPI hydrogel successfully. Additionally, the release and the cell viability results suggest that CBD was still active post-encapsulation, suggesting that WPI has a protective effect post-encapsulation.

With regard to the use of WPI as a hydrophobic drug delivery system, the results suggest that the hydrogels can withstand both pH and enzymatic degradation in stomach conditions, without releasing CBD. Additionally, the release and enzymatic degradation is more pronounced in conditions consistent with the intestines, suggesting higher CBD release in the intestines. When coupled with the result in the cell viability assay, the investigation would conclude that there is the potential in WPI hydrogels to effectively deliver hydrophobic molecules to the target site in CRC, which provides additional evidence supporting WPI-CBD hydrogels as a prime candidate for further analysis for CRC treatment.

## 5. Outlook

The work encompassed in this manuscript focused on the potential of WPI to deliver hydrophobic medication to treat CRC. However, there are still further questions to be answered. These include a characterization of the synthesized hydrogels, elucidation of the water state of the water, differentiation of the influences of hydrophobic and electrostatic interactions on swelling and development of experiments to test the effect of hydrophobic molecules of known hydrophobicity on the swelling potential of the hydrogels.

## Figures and Tables

**Table 1:** Concentration variables of the fabricated WPI-CBD hydrogels.

Sample	% WPI	CBD Concentration (µM)
WPI-CBD0	40	0
WPI-CBD1	40	10
WPI-CBD2	40	20
WPI-CBD3	40	30
WPI-CBD4	40	40
WPI-CBD5	40	50

**Table 2:** The corresponding result for the CBD calibration chart in Figure 4b.

CBD Concentration (μM)	Absorbance at λ273 nm
10	0.17
20	0.36
30	0.53
40	0.69
50	1.04

## Data Availability

The data are contained within the article.
